# Should we abandon the patient-specific instrumentation ship in total knee arthroplasty? Not quite yet!

**DOI:** 10.1186/s12891-021-04581-2

**Published:** 2021-08-24

**Authors:** László Török, Péter Jávor, Petra Hartmann, László Bánki, Endre Varga

**Affiliations:** 1 Department of Traumatology, University of Szeged, Semmelweis u. 6, Szeged, 6725 Hungary; 2 Department of Sports Medicine, University of Szeged, Tisza Lajos Krt. 107, Szeged, 6725 Hungary

**Keywords:** “arthroplasty, replacement, knee”, “Surgical instruments”, “Printing, three-dimensional”, “Patient-specific instrumentation”

## Abstract

Patient-specific Instrumentation (PSI) is an innovative technique aiding the precise implementation of the preoperative plan during total knee arthroplasty (TKA) by using patient-specific guides and cutting blocks. Despite of the theoretical advantages, studies have reported contradictory results, thus there is no consensus regarding the overall effectiveness of PSI. Through the critical assessment of a meta-analysis published lately, this correspondence aims to highlight the complexity of comparing the efficacy of PSI to standard instrumentation (SI). The accuracy of component alignment, patient-reported outcome measures (PROMs), surgery time, blood loss, transfusion rate, and postoperative complications are commonly used outcomes for investigating the efficacy of PSI-aided TKA. By assessing component alignment, the expertise of the surgeon(s) should be taken into consideration, since PSI may not provide benefits for expert surgeons but might improve accuracy and patient safety during the learning curve of novice surgeons. With respect to PROMs and postoperative complications, PSI may not improve short-term results; however, long-term follow up data is missing. Regarding transfusion rates, favorable trends can be observed, but further studies utilizing recent data are needed for a clear conclusion. When assessing surgery time, we suggest focusing on operating room turnover instead of procedure time.

## Main text

To date, there is no consensus regarding the superiority of patient-specific instrumentation (PSI) over standard instrumentation (SI) in performing total knee arthroplasty (TKA). Lately, the meta-analysis of Kizaki et al. [1] underlined lacking benefits of PSI with respect to patient-reported outcome measures (PROMs), surgery time, transfusion rate, and postoperative complications. Although the study has considerable merit, we would like to emphasize the complexity of the issue and shed light on aspects that can provide a basis for the appropriate judgement of the effectiveness of the PSI-aided TKA.

## Choosing the study period

The study of Kizaki et al. included 38 records published between 2012 and 2018; nevertheless, the period of data collection started from 2010 in some studies. Given the dramatic technological development over the past decade, using 8–10-year-old data for the assessment of a surgical method with a software background may not reflect the effectivity of the currently available technology. The approach to software development is much different than a decade ago [2, 3]. Accordingly, there is already a difference between the performances of 1st and 2nd generation PSI designs [4] and using data only from the past 5 years in such a rapidly evolving field would be more reliable.

## Expert vs trainee surgeon

The PSI technology strives for an improved alignment and component position with custom-fit cutting blocks and guides designed preoperatively to fit the patient’s anatomy of the knee [1]. However, expert, high-volume surgeons often have been reported to achieve the same radiological accuracy for TKA with the conventional technique and with PSI [5, 6]; The cutoff number ranges 13–50 procedure yearly for TKA to separate low-volume surgeons from high-volume surgeons [7]. The study of Kizaki et al. reported 336 patients including 29 patients with bilateral lateral femoral bowing (> 5°) receiving TKAs in a 3-year-long study period from the same surgeon. The large number of cases indicates a high-volume surgeon making careful preoperative planning with long films before conventional TKAs, which could hardly be outperformed by using the PSI. Notably, one of the main advantages of the use of PSI that can increase patient safety during the learning curve of novice surgeons, as PSI might allow non-expert surgeons – even with no prior experience- to achieve the same level of accuracy as expert surgeons [5, 6, 8].

## Patient reported outcome measures

Kizaki et al. utilized various PROMs (KSS knee, KSS function, KSS total, Oxford, WOMAC, KOOS symptom, KOOS pain, KOOS ADL, KOOS sports, KOOS QoL, EQ-5D VAS, SF-12 physical score, and SF-12 mental score) to assess and quantify the success rates of PSI and SI. According to their results, PSI did not improve PROMs among patients followed both for less than 1-year and for 1-year or more. However, when evaluating patient satisfaction, the short follow-up time of the included studies should be taken into consideration as most records investigated a postoperative period ranging from 3 to 24 months. Some complications such as aseptic loosening can easily occur in later postoperative phases as well, affecting patient satisfaction and need for revision surgery [9]. Therefore, the long-term influence of PSI on patient satisfaction may worth further investigation.

## Surgery time

Significant difference in surgery time was not found between PSI and SI groups in the meta-analysis of Kizaki et al. Although the study does not paraphrase surgery time, it corresponds to procedure time (from skin incision or torniquet placement) according to the included records. Nevertheless, the main time benefit of PSI is considered to lie in turnover time instead of procedure time, due to the reduced number of instruments and instrument trays [10]. As operating room turnover includes cleaning, and the preparation and replacement of necessary material [11, 12], its association with the number of trays can be presumed. In contrast to the controversy regarding PSI’s procedure time advantage, improving turnover is more clearly supported by clinical data [10, 13, 14]. For this reason, the time-effectiveness of PSI should not be evaluated by taking only procedure time into consideration. Additionally, the time requirement of preoperative planning further complicates the issue.

## Blood loss and transfusion rate

Despite of significantly lower blood loss by PSI, the difference between transfusion rates did not reach significance level according to Kizaki et al. The small effect size, as a potential explanation of the difference in blood loss was mentioned by the authors; however, the heterogeneity of included studies was also considerably high (71%) regarding this parameter. Furthermore, the authors accentuated that most systematic reviews demonstrating the superiority of PSI over SI used surrogate markers such as blood loss as primary outcomes, leading to false conclusion regarding the real advantages of the PSI method. It is important to note that several studies found significantly decreased blood loss with PSI [15–18], and blood loss can influence transfusion requirements. According to this, certain trends could be observed in transfusion rates in the study of Kizaki et al. as well (14% vs 20%). In case of rapidly developing techniques such as PSI, favorable trends may be interpreted as an incentive for further development rather than as a clear lack of benefits.

## Complications

The authors investigated postoperative complication rates (surgical site infection (SSI), deep vein thrombosis (DVT), and need for revision TKA) between the groups and did not find a significant difference. However, the complication rates were small (by PSI TKA: 1.3% for SSI, 1.0% for DVT, and 0.5% for revision TKA), thus it was stated by the authors as well that the pooled events were insufficient to draw a conclusion. Additionally, the short follow-up time of the included studies should be taken into consideration by revision TKA rates. Most records investigated a postoperative period ranging from 3 to 24 months. Maximum follow up was 44 months. According to the literature, aseptic loosening is one of the most common indications for revision TKA, and its cumulative incidence doubles itself from 24 to 48 months postoperatively; moreover, it displays a substantial, continuous growth in the subsequent years as well [9]. Consequently, a study period of at least 48 months would be desirable for the comprehensive investigation of complications. According to this, studies in this issue commonly utilize long follow-up periods (up to 8 years) [19–21].

## Summary and conclusions

**Fig. 1:**
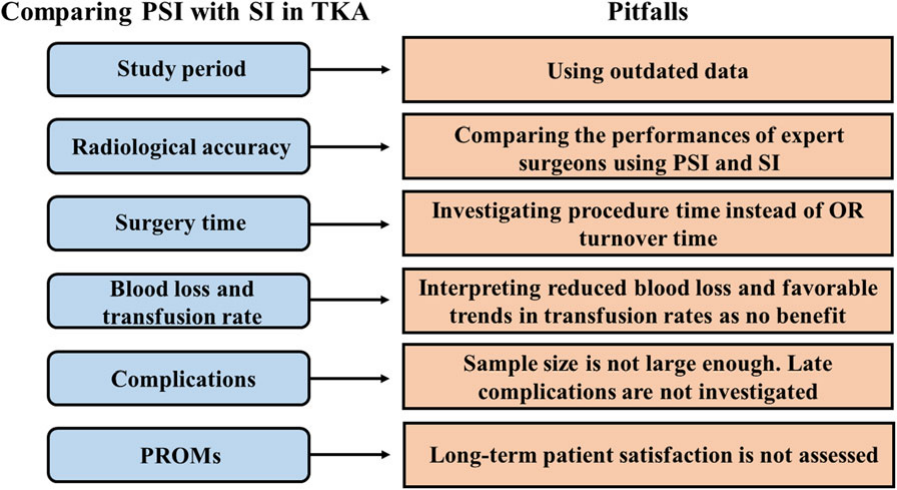
Pitfalls of comparing PSI with SI in performing TKA

In summary, PSI technology for performing TKA develop rapidly, showing promising results from some perspectives and inconsistent from others. Care must be taken to draw the right conclusions from the results of the studies, as the issue is complex and has many facets that may be interpreted differently. A summary diagram highlighting the pitfalls of comparing PSI with SI is presented in Fig. 1.

Additionally, we would like the emphasize that patient-specific techniques and 3D printing are becoming more and more accessible and affordable. Therefore, comprehensive cost-effectiveness analyses should be conducted and repeated in parallel with the development of technology. Ultimately, we suggest further discussion about the utility of PSI and emphasize the need for further research on various patient-specific systems and their long-term effects.

## Data Availability

Our present study uses published data only.

## Abbreviations

PSI: patient specific instrumentation
SI: standard instrumentation
TKA: total knee arthroplasty
PROMs: patient-reported outcome measures
SSI: surgical site infection
DVT: deep vein thrombosis
